# Genome-wide differential expression profiling of mRNAs and lncRNAs associated with prolificacy in Hu sheep

**DOI:** 10.1042/BSR20171350

**Published:** 2018-04-27

**Authors:** Xu Feng, Fengzhe Li, Feng Wang, Guomin Zhang, Jing Pang, Caifang Ren, Tingting Zhang, Hua Yang, Ziyu Wang, Yanli Zhang

**Affiliations:** Jiangsu Livestock Embryo Engineering Laboratory, Nanjing Agricultural University, Nanjing 210095, China

**Keywords:** Hu Sheep, lncRNA, Ovary, Prolificacy, RNA-seq

## Abstract

Reproductive ability, especially prolificacy, impacts sheep profitability. Hu sheep, a unique Chinese breed, is recognized for its high prolificacy (HP), early sexual maturity, and year-round estrus. However, little is known about the molecular mechanisms underlying HP in Hu sheep. To explore the potential mRNAs and long non-coding RNAs (lncRNAs) involved in Hu sheep prolificacy, we performed an ovarian genome-wide analysis of mRNAs and lncRNAs during the follicular stage using Hu sheep of HP (litter size = 3; three consecutive lambings) and low prolificacy (LP, litter size = 1; three consecutive lambings). Plasma luteinizing hormone (LH) concentration was higher in the HP group than in the LP group (*P*<0.05) during the follicular stage. Subsequently, 76 differentially expressed mRNAs (DE-mRNAs) and five differentially expressed lncRNAs (DE-lncRNAs) were identified by pairwise comparison; quantitative real-time PCR (qRT-PCR) analysis of ten randomly selected DE genes (mRNA and lncRNA) were consistent with the sequencing results. Gene Ontology (GO) analysis of DE-mRNAs revealed significant enrichment in immune response components, actin filament severing and phagocytosis. Pathway enrichment analysis of DE-mRNAs indicated a predominance of immune function pathways, including phagosomes, lysosomes, and antigen processing. We constructed a co-expression network of DE-mRNAs and mRNA-lncRNAs, with *C1qA, CD53*, cathepsin B (*CTSB*), *CTSS, TYROBP*, and *AIF1* as the hub genes. Finally, the expression of lysosomal protease cathepsin genes, *CTSB* and cathepsin D (*CTSD*), were significantly up-regulated in sheep ovaries in the HP group compared with the LP group (*P*<0.05). These differential mRNAs and lncRNAs may provide information on the molecular mechanisms underlying sheep prolificacy.

## Introduction

Reproductive ability has important impacts on the profitability of sheep production. Reproduction is a complex process, and traits such as ovulation rate and litter size are genetically affected by causative mutations in some minor and major genes [1,2]. Hu sheep is a local breed in China with high prolificacy (HP), year-round estrus, and an average litter size of 2.06. Approximately 17.35% of these sheep have one lamb, while 17.61% have three lambs. Thus, knowledge of the genes involved in ovulation rate and litter size, and their effects, provides useful information for Hu sheep breeding and for the selection of these traits [3]. To date, mutations in bone morphogenetic protein 15 (*BMP15*), growth differentiation factor 9 (*GDF9*), and bone morphogenetic protein receptor, type 1B (*BMPR-1B*) have been identified in some sheep breeds as fecundity genes that affect follicular development and ovulation [4]. However, mutations in these fecundity genes have different effects on ovulation rate and litter size. For example, *BMP15* has a limited effect on the fecundity of Hu sheep and Merino sheep, but it has a clear effect on the fecundity of Small Tail Han sheep [3,5]. Therefore, it is necessary to identify other major genes affecting Hu sheep prolificacy.

Non-coding RNAs (ncRNAs) play vital roles in eukaryotic gene regulation. Long non-coding RNA (lncRNAs) are greater than 200 nts in length, represent one of the most highly expressed ncRNAs in animals, and regulate the expression of neighboring coding genes [6]. In animals, the level of lncRNA expression is lower than that of normal coding genes, but has more functions than we knew before. Based on its position in the genome, lncRNA can be divided into five categories: divergent lncRNA, sense lncRNA, antisense lncRNA, intergenic lncRNA, and intronic lncRNA [7]. Because of their different modes of action and origin, they are mainly involved in life processes, such as X-chromosome inactivation, chromatin remodeling, histone modification, transcription regulation, and post-transcriptional regulation in the organism [8–11]. In recent years, researchers have focussed on the role of lncRNA in early animal germ cell formation, early embryo implantation and development, and hormone regulation in human and animal reproduction [12–14]. The cumulus cells around bovine oocytes transport large amounts of nutrients and substances, including mRNA and lncRNA, to the oocytes [15]. Furthermore, similar studies in mice have also found that ovarian somatic cells transfer RNA and other cytoplasmic substances into the oocyte, including lncRNA. [16–18]. The lncRNA nuclear paraspeckle assembly transcript 1 (*Neat1*) was found to be essential for corpus luteum formation, and for the establishment and maintenance of female pregnancy in animals [19]. A recent study also showed that the differential regulation of miRNAs and lncRNAs might be related to fecundity in Small Tail Han sheep and Dorset sheep [20]. Despite these findings, research on prolificacy-associated lncRNA in Hu sheep and its target mRNA, especially their interaction networks, remains limited.

In the present study, we compared the patterns of reproductive hormones and *BMPR-1B* mutations in Hu sheep with high and low prolificacy (LP), and then used strand-specific RNA sequencing (ssRNA-seq) to identify the role of mRNAs and lncRNAs in sheep ovaries during this process. We identified genome-wide differentially expressed mRNAs (DE-mRNAs) and lncRNAs (DE-lncRNAs) in each comparison, and Gene Ontology (GO) and Kyoto Encyclopedia of Genes and Genomes (KEGG) enrichment analyses of DE-mRNAs and target genes of lncRNAs were conducted. We further constructed mRNA–mRNA and lncRNA–mRNA regulatory networks associated with sheep prolificacy.

## Materials and methods

### Animals and sample collection

All experimental procedures were conducted in strict compliance with the recommendations of the Guide for Animal Experiments of Nanjing Agricultural University, China (approval ID: SYXK2011-0036).

Ewes with three lambing records were divided into two groups: an HP group (*n*=4, litter size = 3) and an LP group (*n*=4, litter size = 1). Animals were raised at the Taizhou Hailun Sheep Industry Co., Ltd (Jiangsu, China) under similar conditions with free access to feed and water. First, we conducted synchronous estrus before the experiment, a vaginal sponge was implanted for 11 days, followed by the administration of 0.2 mg cloprostenol at the time of sponge removal. Estrus was tested by the ram at 9 a.m., 12 noon, and 6 p.m. each day. After the first estrus was detected, blood was collected from the jugular vein at 9 a.m. every morning, and the ewes were used for intensive blood collection prior to slaughter, when they were confirmed to be in estrus. All blood samples were treated with heparin sodium for anticoagulation and centrifuged at 3000 rpm for 15 min to separate the plasma, which was then stored at −20°C. After slaughter, all left ovary samples were immediately collected and stored at −80°C for total RNA extraction.

### Polymorphism analysis of BMPR-1B

To determine the type of mutation in the *BMPR-1B* gene in the HP and LP groups, blood was collected from all the ewes. Exon 6 (741–936 bp) of *BMPR-1B* was amplified. Primer sequences are provided in Supplementary Table S1. PCR was performed in a 40-μl volume containing approximately 20 pmol primer. Amplification conditions were as follows: initial denaturation at 94°C for 5 min; followed by 35 cycles of denaturation at 94°C for 30 s, 60°C for 30 s, extension at 72°C for 30 s; with a final extension at 72°C for 7 min using a T100 Thermal Cycler (Bio–Rad, U.S.A.). The sense strand of the amplified product was subsequently sequenced.

### ELISA

Serum levels of estradiol (E2), follicle stimulating hormone [21], and luteinizing hormone (LH) were assayed using ELISA. Frozen serum samples were thawed slowly at room temperature, along with the assay kits (Kmaels, China, E2: #DRE-S9105c, FSH: #DRE-S0622c, LH: #DRE-S5741c). Samples, standards, and HRP-labeled detection antibodies were sequentially added to the coated microwells precoated with sheep hormones capture antibody, incubated and washed thoroughly. Tetramethyl benzidine (TMB) was converted into blue by peroxidase catalysis and the final yellow color under acidic conditions. The absorbance (OD value) was measured with a microplate reader at a wavelength of 450 nm to calculate the concentration of hormones. All assays were performed according to the manufacturer’s protocol in duplicate.

### RNA extraction, library construction, and RNA-seq

Total RNA was extracted from ovaries using TRIzol reagent (Invitrogen, Carlsbad, CA). RNA concentration and purity were measured using the NanoDrop 2000 spectrophotometer (Thermo Fisher Scientific, Wilmington, DE). RNA integrity was assessed using the RNA Nano 6000 Assay Kit of the Agilent Bioanalyzer 2100 System (Agilent Technologies, CA, U.S.A.). In total, 1.5 μg RNA per sample was used as the input material for rRNA removal using the Ribo-Zero rRNA Removal Kit (Epicentre, Madison, WI, U.S.A.).

Sequencing libraries of six samples (HP group, *n*=3; LP group, *n*=3) were generated using NEBNextR Ultra™ Directional RNA Library Prep Kit for IlluminaR (NEB, U.S.A.) following the manufacturer’s recommendations, and index codes were used to label the sequences of each sample. To select fragments of 150–200 bp, the library fragments were purified with AMPure XP Beads (Beckman Coulter, Beverly, U.S.A.). Then, 3 μl USER Enzyme (NEB, U.S.A.) was used with size-selected, adaptor-ligated cDNA at 37°C for 15 min before PCR. Then, PCR was performed with Phusion High-Fidelity DNA polymerase, Universal PCR primers and Index (X) Primer. Finally, PCR products were purified (AMPure XP system, Beckman Coulter, Beverly, MA, U.S.A.) and library quality was assessed on the Agilent Bioanalyzer 2100 and via quantitative real-time PCR (qRT-PCR). Index-coded samples were clustered on acBot Cluster Generation System using TruSeq PE Cluster Kitv3-cBot-HS (Illumia) according to the manufacturer’s instructions. After cluster generation, the library preparations were sequenced on an Illumina Hiseq platform and then paired-end reads were generated.

### Read mapping and lncRNA prediction

Raw data (raw reads) in fastq format were processed through in-houseperl scripts. In this step, clean data (clean reads) were obtained by removing reads containing adapters, reads containing poly-N, and low-quality reads from raw data. At the same time, Q20, Q30, GC-content, and sequence duplication of the clean data were calculated. All downstream analyses were based on high-quality clean data. The transcriptome was assembled using Cufflinks (version 2.1.1) and Scripture based on reads mapped to the *Ovis aries* reference genome (Oar_v3.1). The assembled transcripts were annotated using the Cuffcompare program from the Cufflinks package. Unknown transcripts were used to screen for putative lncRNAs. Four computational approaches, including the coding potential calculator (cpc), coding-non-coding index (cnci), protein families database (pfam), and coding-potential assessment tool (cpat) [22–25], were combined to sort non-protein-coding RNA candidates from putative protein-coding RNAs in the unknown transcripts.

Putative protein-coding RNAs were filtered using a minimum length and exon number threshold. Transcripts with lengths exceeding 200 nts, and with more than two exons, were selected as lncRNA candidates, and were further screened using cpc/cnci/pfam/cpa, which can distinguish protein-coding from non-coding genes. Then, the different types of lncRNAs, including lincRNA, intronic lncRNA, and antisense lncRNA, were selected using cuffcompare. Cuffdiff (version 2.1.1) was used to calculate fragments per kilobase of exon per million fragments mapped (FPKMs) of coding genes and lncRNAs in each sample [26]. Gene FPKMs were computed by summing the FPKMs of transcripts in each gene group.

Based on the mode of lncRNA action on target genes, we used two predictive methods. First, lncRNA regulates the expression of neighboring genes, which can be predicted based on the position of lncRNA and mRNA, and adjacent genes within a range of 100 kb of its target gene. Second, lncRNA and mRNA function through base pairing, and the LncTar [27] target gene prediction tool was used to predict the target gene of lncRNA.

### Differential expression analysis and gene functional annotation

Differential expression analysis of DE-mRNA and DE-lncRNA in the two groups was performed using the DESeq R package (1.10.1). DESeq provides statistical routines to determine differential gene expression within digital data using a model based on the negative binomial distribution. The resulting *P*-values were adjusted using the Benjamini and Hochberg approach to control the false discovery rate (FDR). Genes with an adjusted FDR < 0.05 and absolute value of log_2_ (fold change) >1 found by DESeq were classed as being differentially expressed. Gene function was annotated based on the following databases: Nr, Pfam, Swiss-Prot, KEGG, GO. GO enrichment analysis of the differentially expressed genes (DE-mRNAs) was implemented by the topGO (R package, version 2.8) R package. We used KOBAS [28] software to test the statistical enrichment of differentially expressed genes in the KEGG pathways.

### Construction of mRNA–mRNA and lncRNA–mRNA networks

To infer the function of DE-lncRNA and DE-mRNAs in sheep prolificacy, we constructed a complementary pair network based on mRNA and mRNA as well as between mRNA and lncRNA, by using cytoscape (V3.4.0).

### Validation of gene expression of by qRT-PCR

For the qRT-PCR analysis, 1 μg total RNA was reverse transcribed using RT reagent kits with gDNA Eraser (Takara, China) according to the manufacturer’s protocol. Real-time PCR was performed on an ABI 7300 (Applied Biosystems, Foster City, CA, U.S.A.) with Fast Start Universal SYBR Green Master (ROX) (Roche, Mannheim, Germany). The following program was used: 95°C for 5 min, followed by 40 cycles at 95°C for 10 s, 60°C for 30 s, and 72°C for 30 s. Primers for mRNAs and lncRNAs are shown in Supplementary Table S1. Glyceraldehyde-3-phosphate dehydrogenase (GAPDH) was used as an internal reference to normalize target gene expression. All experiments were performed in triplicate.

### Immunohistochemistry assay of cathepsin B and cathepsin D

Ovaries of ewes were fixed in 4% paraformaldehyde, then embedded in paraffin. Paraffin sections were dewaxed in xylene and subsequently gradually hydrated by gradient alcohol, finally transferred to water. The dewaxed sections incubated with 3% H_2_O_2_ at 37°C for 15 min to quench the endogenous peroxidase, and antigen retrieval was carried out in citrate buffer solution at 100°C for 15 min. After cooling down to room temperature, the sections were blocked by 5% BSA at 37°C for 30 min; then incubated the sections at 4°C for 12 h with anticathepsin B (#ab125067,abcam, rabbit monoclonal, IgG) and anticathepsin D (#orb180468, Biorbyt, goat polyclonal, IgG) primary antibody. Sections were washed with PBS, then incubated with corresponding secondary antibody and stained by using rabbit IgG SABC immunohistochemical staining kit (#SA2002, Boster Biological Technology Co. Ltd, China) and goat IgG SABC immunohistochemical staining kit (#SA2003, Boster Biological Technology Co. Ltd, China). All sections were examined under microscope (Nikon, Japan).

### Statistical analyses

All data are presented as the mean ± S.E.M. When comparisons were made, an independent-sample *t* test was performed using SPSS 24.0 software (SPSS Inc., Chicago, IL, U.S.A.), and *P*<0.05 was considered statistically significant.

## Result

### Analysis of plasma reproductive hormone concentration and BMPR-1B polymorphism

**Figure 1:**
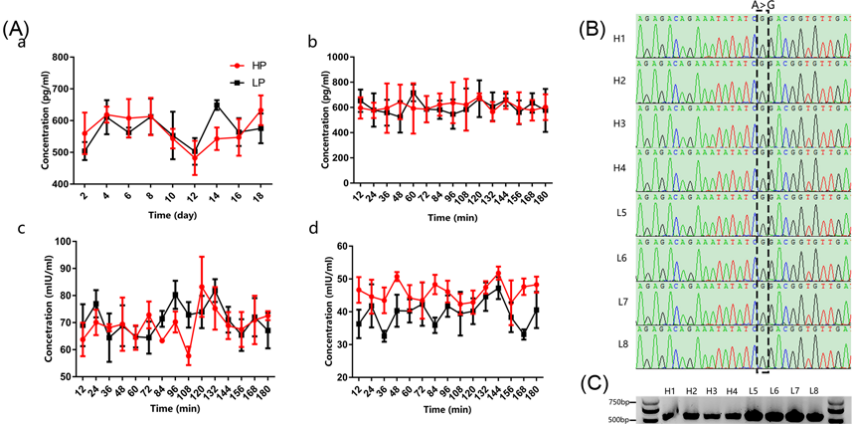
Changes in the concentrations of reproductive hormones and *BMPR-1B* mutations in HP and LP Hu sheep (**A**) The black and red lines represent data for the HP and LP Hu sheep, respectively. (**a**) Changes in the E2 concentration during the estrous cycle; (**b**) changes in the E2 concentration during intensive blood collection; (**c**) changes in the FSH concentration during intensive blood collection; (**d**) changes in the LH concentration during intensive blood collection. (**B**) Sequencing results for eight ewes. In eight ewes, the CAG site in exon 6 of BMPR-1B amplification products were CGG. (**C**) Representative PCR for the *BMPR-1B* gene performed on samples of every ewe.

Plasma E2, FSH, and LH concentrations are shown in Figure 1A. Considering the existence of time variables, we performed repeated measures analysis of covariance (RMANCOVA) to compare the difference in peripheral blood hormone levels between HP ewes and LP ewes. The results show that there were no significant differences in E2 levels during the estrous cycle between HP and LP Hu sheep (*P*>0.05). Additionally, there were no significant differences in the pattern of change in FSH levels between these two groups prior to ovulation (*P*>0.05). However, compared with the LP group before ovulation, the plasma level of LH was significantly higher in the HP group (*P*<0.05).

The mutated sites in BMPR-1B were examined and sequenced (Figure 1B). One genotype, BB, was detected in all the eight ewes. The nucleotide sequence obtained from the BB genotype was identical with that obtained from the wild-type ++, except for an A→G transition at base 746 in the coding region of the *BMPR-1B* gene. This mutation resulted in an amino acid change from glutamine in the wild-type to arginine in the BB genotype (CAG→CGG, Q249R) (Figure 1C).

### Transcript assembly and quality control

**Figure 2:**
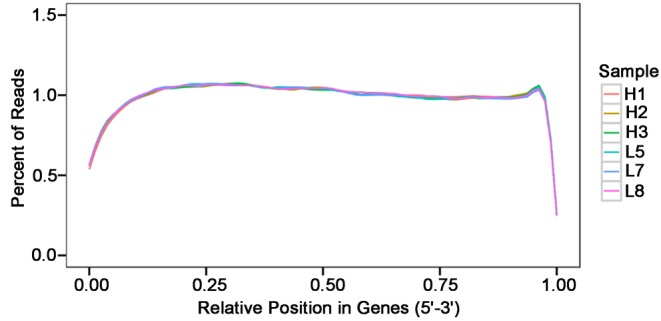
The positional distribution of mapped reads on mRNA The abscissa is the normalized mRNA position and the ordinate is the percentage of reads in the total range of the corresponding mapped reads. As the length of the reference mRNA differs, each mRNA is divided into 100 intervals by length, and the number and percentage of mapped reads in each interval are counted. The figure summarizes the proportion of mapped reads.

In total, 238325161 raw paired-end reads were obtained from six samples. In order to test the quality of RNA-seq data, we performed a series of quality control analyses. First, the Q30 of reads in all samples ranged from 93.86 to 94.22%. The average GC content of six libraries was 50.13%. Next, we examined the total coverage of reads from the 5′ to 3′ end of genes. We found that in all samples, RNA-seq reads were evenly distributed, with the exception of the 5′ and 3′ ends (Figure 2). These findings suggest that the sequencing data were highly reliable (Table 1). The mapping rate to the *O. aries* reference genome (Oar_v3.1) of our clean data was between 80.95 and 82.17% (Table 2).

**Table 1:** Sample quality data assessment statistics

Sample	Obtained reads	Obtained bases (G)	Q30 (%)	GC (%)
H1	41464876	12336861648	94.19	49.87
H2	33523161	9953393728	93.86	49.47
H3	39575613	11747175606	94.07	48.6
L5	42853663	12718293562	94.15	51.23
L7	36307176	10778562464	94.22	51.32
L8	44600672	13235569958	94.09	50.29

Abbreviations: GC (%), sample GC content; Obtained bases, the number of bases obtained; Obtained reads, the number of reads obtained; Q30 (%), percentage of bases with a mass value greater than or equal to 30.

**Table 2:** Sequence comparison of sample sequencing data with the selected reference genome

Sample	Total reads	Mapped reads	Uniq mapped reads	Multiple mapped reads	Reads map to ‘+’	Reads map to ‘–’
		67460493	63602603	3857890	34794402	32666091
H1	82929752	(81.35%)	(94.28%)	(5.72%)	(41.96%)	(39.39%)
		54271781	51334082	2937699	28090839	26180942
H2	67046322	(80.95%)	(94.59%)	(5.41%)	(41.90%)	(39.05%)
		65034922	61540449	3494473	33600115	31434807
H3	79151226	(82.17%)	(94.63%)	(5.37%)	(42.45%)	(39.71%)
		70080879	65899898	4180981	36079700	34001179
L5	85707326	(81.77%)	(94.03%)	(5.97%)	(42.10%)	(39.67%)
		59625107	56228566	3396541	30705660	28919447
L7	72614352	(82.11%)	(94.30%)	(5.70%)	(42.29%)	(39.83%)
		72211452	67737688	4473764	37246343	34965109
L8	89201344	(80.95%)	(93.80%)	(6.20%)	(41.76%)	(39.20%)

### Genome-wide identification of DE-mRNAs and DE-lncRNAs

**Figure 3:**
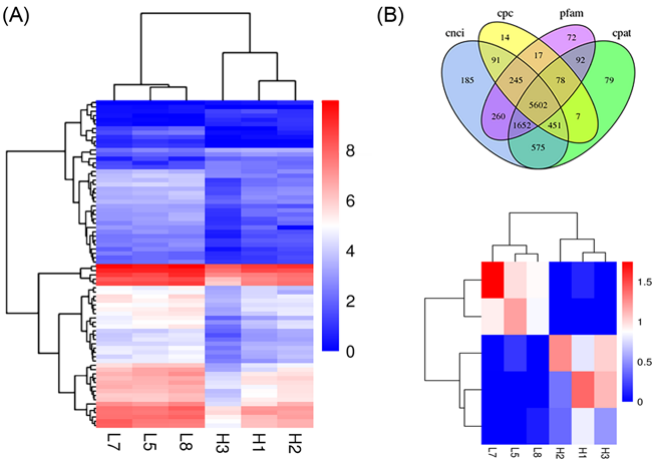
DE-mRNA and lncRNA (**A**) Hierarchical cluster showing relative expression levels of 76 mRNAs between two groups; (**B**) non-coding transcripts identified by the four predictors were analyzed statistically, and then a Venn diagram of all predicted results was obtained. The lncRNA was predicted by four methods and 5602 lncRNA were found. The hierarchical clusters show the relative expression of five lncRNAs between two groups.

For further analysis, 76 DE-mRNAs were identified between HP and LP Hu sheep (Figure 3A), 66 of which were up-regulated and 10 of which were down-regulated, with 4 identified as new genes in sheep (Supplementary Table S2). Based on the results of the comparison, 2424 new genes were discovered including 1529 functional annotations, by analyzing alternative splicing predictions, optimizing gene structure, and exploring new genes. Furthermore, 5602 lncRNAs were obtained using four prediction methods, and 5 were found to differ significantly between the two groups (*P*<0.05) (Figure 3B). The hierarchical clusters of the DE-mRNA and DE-lncRNA revealed the expression patterns in HP and LP ewes (Figure 3A,B).

### Validation of RNA-seq data by real-time PCR

**Figure 4:**
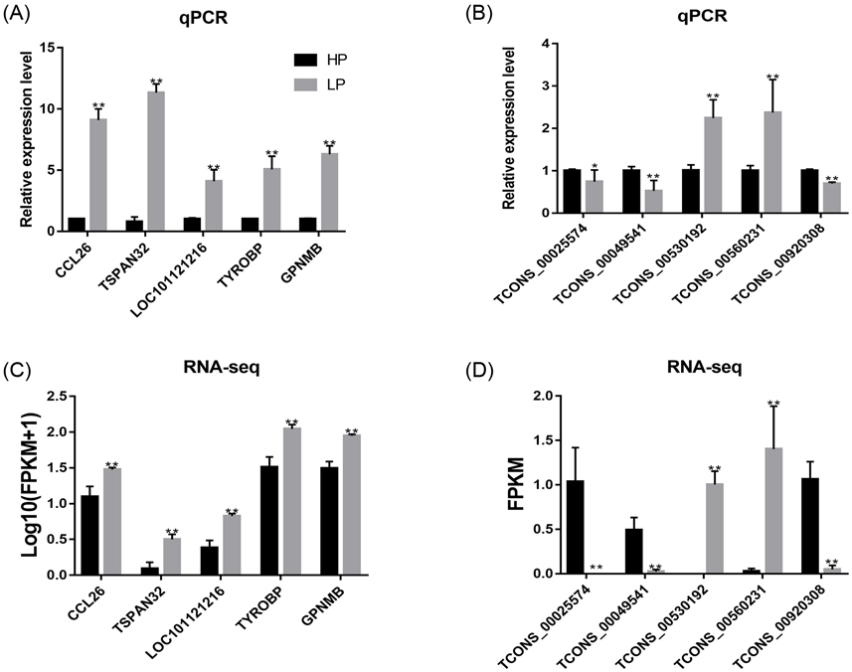
Validation of RNA-seq results using qRT-PCR and RNA-seq, respectively (**A**,**B**) The relative expression of DE-mRNA and DE-lncRNA was determined by q-PCR. Comparisons by independent-sample *t* test, using SPSS 24.0. *: *P*<0.05; **: *P*<0.01. (**C**) RNA-seq data of DE-mRNA; relative expression level, normalized by log_10_ (FPKM + 1). (**D**) RNA-seq data of DE-lncRNA; relative expression level, normalized by FPKM. Abbreviations: CCL26, C–C motif chemokine ligand 26; *GPNMB*, glycoprotein nmb*; LOC101121216*, serum amyloid A protein-like; *TSPAN32*, tetraspanin 32; *TYROBP*, TYRO protein tyrosine kinase binding protein.

To validate the RNA-seq data, we selected five DE-mRNAs and five DE-lncRNAs and determined the expression of these RNAs by real-time PCR (Figure 4). The expression of each mRNA or lncRNA was examined in the HP and LP groups, and the results were consistent with those obtained by sequencing. Primers of mRNA and lncRNA are shown in Supplementary Table S1.

### Functional annotation and enrichment analysis

**Figure 5:**
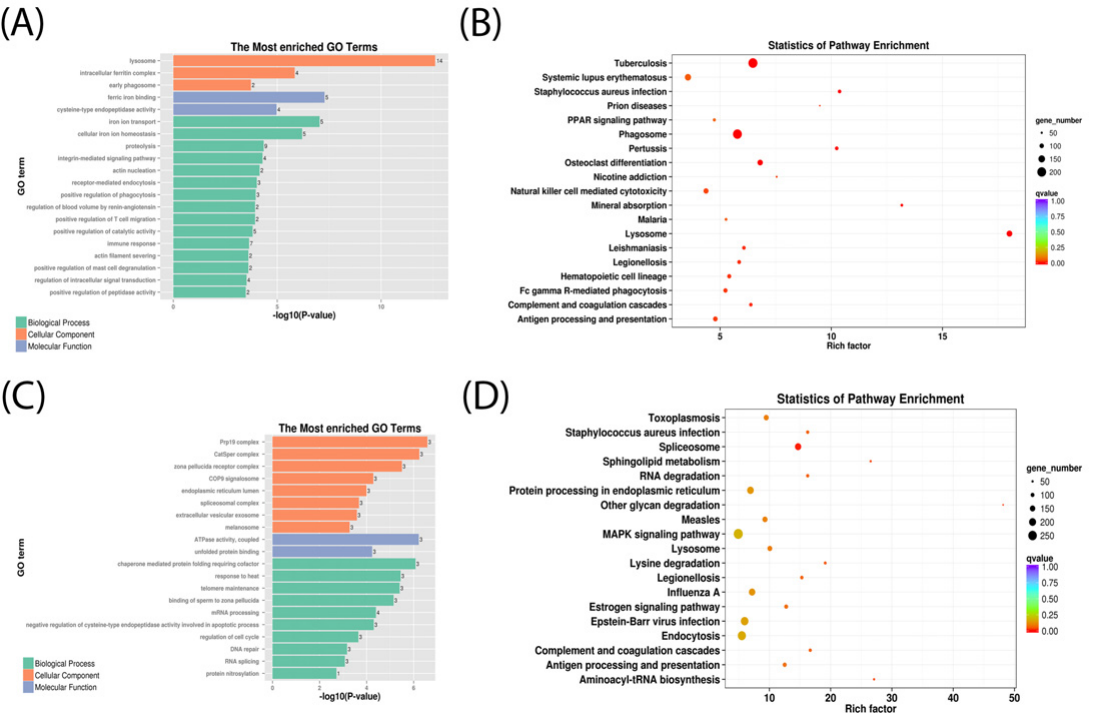
Top GO and KEGG pathway enrichment analyses of DE-mRNAs and target genes of DE-lncRNAs (**A**) Top 20 GO terms of DE-mRNAs. (**B**) Top 20 pathways of DE-mRNAs. (**C**) Top 20 GO terms of the target genes of DE-lncRNAs. (**D**) Top 20 pathways of the target genes of DE-lncRNAs.

To further elucidate the functions of the DE-mRNAs and DE-lncRNAs, GO enrichment analysis was performed using topGO to search the most significant GO term of DE-mRNAs and the target genes of DE-lncRNAs. All of these were assigned to biological processes, cellular components, and molecular function, respectively. Moreover, organ morphogenesis, tissue development, immune system process, regulation of reproductive hormone biosynthetic process, and positive regulation of endothelial cell apoptotic process were identified as significantly enriched GO terms in DE-mRNAs (KS < 0.05, KS: *P*-value of Kolmogorov–Smirnov test, the smaller the KS value, the more significant the enrichment) (Supplementary Tables S3–S5). We also found that most DE-mRNAs were assigned to the GO terms of biological processes (Figure 5A). The targets of DE-lncRNAs were mostly enriched in immune system process, cell differentiation, and tissue development. The most enriched GO terms for DE-mRNAs and target genes in each comparison are shown in Figure 5A,C, respectively. According to the KEGG analysis, 60 (DE-mRNAs) and 6 (target gene of DE-lncRNAs) were assigned to 33 and 19 pathways, respectively. The most enriched pathway of DE-mRNA and target genes of DE-lncRNA are shown in Figure 5B,D. They are predominantly associated with immune functions, such as phagosomes, lysosomes, and antigen processing and presentation, by DE-mRNAs (*P*<0.05).

### Protein co-expression network and lncRNA–mRNA interaction network

**Figure 6:**
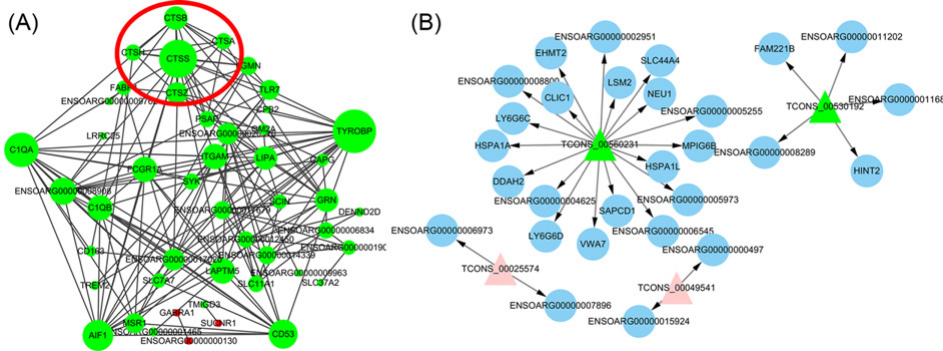
Network between DE-mRNAs and DE-lncRNAs (**A**) Using STRING, the network of mRNA was constructed with 48 DE-mRNAs, and a significant interaction between these genes was found. Green nodes represent up-regulated genes, pink nodes represent down-regulated genes. The size of the node represents the number of genes that interact with it, the larger the node, the more genes that interact with it. Amongst the red circles is a member of the cathepsins family such as *CTSB*. (**B**) Four DE-lncRNAs were used to construct a network between lncRNA and mRNA. The nodes of the triangle represent lncRNA, and the circular nodes represent mRNA. Green nodes represent up-regulated genes, pink nodes represent down-regulated genes.

To better illustrate the relationships between mRNAs and lncRNA, we constructed two co-expression networks (Figure 6A). Co-expression networks cluster multiple transcripts into functional modules based on the correlations with gene expression. We selected 48 DE-mRNAs to construct functional networks by referring to the STRING database, with each gene corresponding to a node. Two genes are connected by an edge, indicating a strong correlation (i.e. either positive or negative). Within the network analysis, we focussed on genes that interact with five more other genes; *C1qA, CD53*, cathepsin B (*CTSB*), *CTSS, TYROBP*, and *AIF1* were hub genes in the network. In order to understand the effect of lncRNA on the regulation of lnRNA, we constructed an mRNA–lncRNA regulatory network, which consisted of nodes, including 28 mRNAs and four lncRNAs (Figure 6B). These lncRNAs were predicted to regulate their targets by a *trans*-action mode.

### Expression pattern of CTSB and CTSD in ovaries

**Figure 7:**
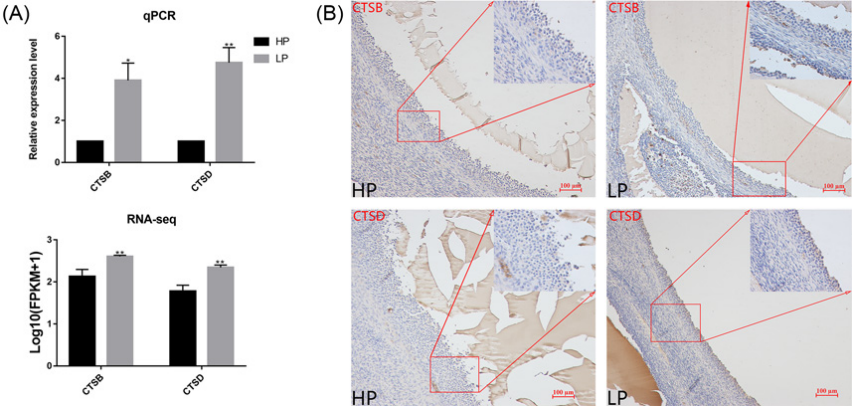
The pattern of *CTSB* and *CTSD* expression in the ovaries (**A**) The relative expression level of *CTSB* and *CTSD* as determined by qRT-PCR, and the RNA-seq data of *CTSB/CTSD* relative expression, normalized by log_10_ (FPKM + 1). Comparisons were made using independent-sample *t* test, with SPSS 24.0. *: *P*<0.05; **: *P*<0.01. *CTSB* and *CTSD* primers are shown in Supplementary Table S1. (**B**) Representative immumohistochemical staining for *CTSB* and *CTSD* in the ovaries of HP and LP ewes. *CTSB* and *CTSD* proteins were both positively expressed in granulosa cells.

In the present study, we found that most DE-mRNAs were enriched in the lysosome pathway. Two proteases, CTSB and CTSD, are involved in the function of lysosomes, and their genes are differentially expressed between HP ewes and LP ewes. Both the *CTSB* and *CTSD* genes are expressed in granulosa cells, and their expression are regulated by LH concentration [29]. The results obtained using peripheral blood from HP ewes revealed that the LH concentration was higher than that from LP ewes. Notably, the high LH concentration in HP ewes could reduce the expression of *CTSB* and *CTSD* in ovaries compared with that in LP ewes. Therefore, we hypothesized that CTSB and CTSD play very important roles in granulosa cells before ovulation, and may influence the litter size in ewes. In order to determine the expression pattern of *CTSB* and *CTSD* in sheep ovaries, we performed qRT-PCR and immumohistochemical staining. Similar to the results obtained with RNA-seq, the expression of *CTSB* and *CTSD* was significantly (*P*<0.01) higher in LP ewes than in HP ewes (Figure 7A). For immumohistochemical staining, we found that both *CTSB* and *CTSD* were expressed in the granulosa cells of mature follicles (>2.5 mm) of Hu sheep ovaries, as reported in other animals [30–32] (Figure 7B).

## Discussion

Mammalian genomes encode thousands of lncRNAs. lncRNAs have gained widespread attention due to their roles in gene regulatory networks, and in a wide range of biological processes [33,34]. To date, many studies have associated the dysregulation of lncRNAs with reproduction, including germ cell formation, early embryo implantation and development, and reproductive hormone regulation. The present study examined the expression profile of mRNAs and lncRNAs in sheep ovaries associated with prolificacy, and found that mRNAs and lncRNAs were differentially expressed in the different groups analyzed. Further analyses of the interaction networks of mRNAs and lncRNA indicated that these DE-mRNA and lncRNA expression profiles might play key roles in sheep prolificacy.

The levels of E2, FSH, and LH are critical for follicle development and ovulation, and their effects on ovaries will ultimately affect litter size. The reproductive cycle of sheep usually includes three or four follicular waves, and the occurrence of follicular waves is largely controlled by dynamic changes in FSH levels, the diameter of a mature follicle affects the reactivity of the ewes to FSH. When sheep’s reactivity to FSH is reduced, the number of follicular waves will reduce, resulting in low ovulation rates. [35]. E2 levels in the ewes were similar during the estrous cycle and in pre-ovulation, suggesting that ovarian regulation by E2 is not the main reason for the difference in litter size [36]. Thus, the E2 levels of two groups were measured during the estrous cycle and during pre-ovulation; however, the results revealed no significant differences between the two groups of ewes. FSH in synergy with LH stimulates follicle maturation and ovulation. FSH and LH play important roles in follicular development [37]. A high FSH/LH ratio during the early stage of follicular development promotes primordial follicle entry into the pre-antral follicle [38]. The lower the ratio of FSH/LH before ovulation, the greater the litter size [39]. However, in our present study, there were no significant differences in the plasma concentrations of E2 and FSH between the HP and LP groups, but the ratio of FSH/LH in HP ewes was significantly lower than that in LP ewes, which might result in higher litter size in the HP ewes. In addition, by amplifying the mutation sites in BMPR-1B, we found that the *FecB* genes of the eight experimental ewes were identical, and were all of the BB genotype. Therefore, in addition to the BB genotype mutation of BMPR-1B, other important factors may affect litter size in sheep.

In the present study, 76 DE-mRNAs and 5 DE-lncRNAs were identified by pairwise comparison. qRT-PCR analysis revealed that the results correlated well with the RNA-seq data. Using GO enrichment analysis, we identified some GO terms related to the immune response and inflammatory response, such as immune system process, antigen processing and presentation, and positive regulation of interleukin-1β secretion (Supplementary Table S3). These results may indicate that the immune-like response that occurs before ovulation, approaching the LH peak, might affect the litter size of Hu sheep. Additionally, some GO terms related to reproductive hormone metabolism were found in our study, including regulation of testosterone biosynthetic process, progesterone metabolic process, androsterone dehydrogenase (B-specific) activity, and dihydrotestosterone 17-β-dehydrogenase activity; both these hormones were affected by LH [40] (Supplementary Tables S3 and S5). Conversely, the GO terms lysosomal membrane and positive regulation of endothelial cell apoptotic process were associated with apoptosis of ovarian cells, including granulosa cells. Using KEGG pathway enrichment analysis, we were unable to identify the pathway directly associated with reproduction; however, the antigen processing and presentation, and natural killer cell mediated cytotoxicity pathways are involved in the immune response (Supplementary Table S8). GO and KEGG enrichment data indicated that genes involved in the immune response and those associated with lysosomes might play important roles in ovulation. Changes in the expression of *C1q, TLRs*, and *cathepsin* genes that associated with the immune response were also found in other investigators’ studies of ovulation, it was similar to our study [41].

In recent years, ovulation has been described as a complex process involving an inflammatory and immune response, which includes follicular development, final follicle rupture, and the release of oocytes, followed by the formation of the corpus luteum [42–44]. Ovulation can up-regulate the expression of genes related to the apoptosis of follicular cells, and can then lead to physiological damage of the follicles. However, increased plasma LH concertation may counteract this damage through the differential expression of some mRNAs and lncRNAs between HP and LP ewes. Allograft inflammatory factor 1 (AIF-1) is involved in macrophage activation, and is constitutively expressed in monocytes and macrophages. Small populations of macrophages are found in the ovaries, and these are essential for tissue homeostasis and normal ovarian function. The number of macrophages varies depending on the stage of the ovarian cycle, but these cells are mostly associated with the corpus luteum and follicular atresia [45]. Macrophages resident in the Graafian follicles have great influence on ovulation and corpus luteum formation [46]. As a class of transmembrane recognition receptors, TLRs regulate the immune response by stimulating the release of cytokines during inflammation-like processes [47]. Up-regulation of TLR7 has been found in the ovaries of LP ewes, and in the membranes of intracellular endosomes and lysosomes, where it is involved in lysosomal function [48]. Furthermore, the pathway related to TLRs directly impacts granulosa cell function by controlling steroidogenesis and interacting with FSH signaling [49]. Moreover, *TLR* gene expression could be regulated by gonadotropins [50]. In the present study, cathepsin genes (*CTSA, CTSB, CTSD, CTSH, CTSS, CTSZ*) and other lysosome-related genes (*lipase A*, lysosomal acid type, *LIPA*; legumain, *LGMN*) were up-regulated in LP ewes. The cathepsin family includes proteolytic enzymes that break down many proteins and possess a broad range of proteolytic properties. This family of proteases is mainly found in lysosomes, cytoplasm, and endosomes [51]. To delineate the differential expression patterns, mouse cathepsins B, K, S, and Z were reported to be expressed in developing oocyte and granulosa cells [31]. The abundance of CTSB, S, K, and Z mRNA in cumulus cells may be related to oocyte quality [32], Therefore, we speculated that the higher expression of CTSB, S, and Z may be one explanation for the low prolificacy of LP ewes. CTSB and CTSD are two common cathepsins, which are involved in apoptosis. Both these proteins are regulated by LH [52]. CTSB is involved in the regulation of apoptosis during serum-starvation in follicles, which suggests that CTSB might play a regulatory role in follicular apoptosis [30]. Conversely, reactive oxygen species (ROS) is also a necessary signal for ovulation, which may be triggered by LH [53]. ROS are key signaling modules involved in the initiation of apoptosis in antral follicles, and granulosa cells of antral follicles [54]. LH surge increases ROS levels, which can lead to the release of lysosomal enzymes, such as CTSB and CTSD, subsequently triggering apoptosis [55,56].

Previous studies have shown that mRNAs and lncRNAs may be involved in ovarian function and follicular development, thus regulating the fecundity of female animals [57,58]. We constructed a network diagram of lncRNAs and mRNAs to determine the regulation of mRNA by lncRNA. We identified some target genes, such as heat shock protein family A (Hsp70) member 1 like (HSPA1L), Hsp70 member 1A (HSPA1A), and histidine triad nucleotide binding protein 2 (HINT2), which are involved in apoptosis. HSPA1L and HSPA1A are members of the HSP70 family, and the HSP70 family is involved in lysosomal stability [59]. Once the lysosomal structure is unstable, it will lead to protease leakage, and then to apoptosis [56]. HSPA1 can block apoptosis by inhibiting BAX activation. In addition, HSPA1 can interact directly with the mitochondrial pathway, which may also block apoptosis [21]. HSPA1L and HSPA1A are predicted to be target genes for lncRNA *TCONS_00560231*, suggesting that lncRNA *TCONS_00560231* may also be involved in the regulation of granulosa cell apoptosis. As a member of a superfamily of histidine triad hydrolases, *HINT2* has been found to affect mitochondria-dependent apoptosis in hepatocytes [60]. *HINT2* is expressed in the mitochondria of adrenal cortical cells and is involved in the steroidogenic response of calcium-dependent and calcium-independent agonists. Calcium-dependent actions of *HINT2* on steroidogenesis may be associated with their ability to maintain mitochondrial potential. Moreover, *HINT2* affects the basic metabolism of certain nucleotides, which may play an important role in the activation of protein kinases and the interaction with certain transcription factors in mitochondrial metabolism. Thus, *CTSD* and *CTSB* may activate mitochondria-dependent apoptosis in granulosa cells [56], having the same effect as lncRNA *TCONS_00530192* regulated *HINT2. TCONS_00530192* may be involved in granulocyte apoptosis by regulating *HINT2*.

Generally, we found that the BB-type mutation in BMPR-1B may represent only one factor limiting increased litter size. LH levels in HP ewes were significantly higher than those in LP ewes before ovulation. The difference in LH concentration in the peripheral blood during ovulation might result in the differential expression of mRNA and lncRNA in the ovaries of HP and LP ewes. Ovulation is considered to be an inflammatory response. In this process, some inflammation-related genes, such as *AIF1, TYROBP, SLC11A1*, and *PYCARD*, and some immune-related genes, such as *TLR7, CCL26, C1qA*, and *C1qB* are up-regulated. The high expression of these genes leads to increased expression of lysosomal-related genes, such as *CTSD* and *CTSB*, which may be closely related to apoptosis. *CTSS, CTSB*, and *CTSZ* are also regulated by LH and are used as markers to assess oocyte quality [32,61]. Moreover, some lncRNAs can regulate their target genes in these reactions. For example, *TCONS_00560231* and *TCONS_00530192* may participate in granulosa cell apoptosis by regulating the *HSPA1A, HSPA1L*, and *HIT2*.

In the present study, we compared the levels of reproductive hormones before ovulation and the BMPR-1B polymorphism between HP and LP Hu sheep. Importantly, we showed the differential mRNA and lncRNA expression profiles associated with sheep prolificacy and constructed interaction networks amongst lncRNAs and mRNAs. Our study lays a solid foundation that may aid in elucidating the regulatory mechanisms of mRNAs and lncRNAs in sheep.

## Supporting information

**Table S1:** Details of primer sequences, expected product sizes and Genbank accession numbers of genes used for qRT-PCR

**Table S2:** The annotation of all DE-mRNAs

**Table S3:** The annotation of all DE-mRNAs

**Table S4:** Top20 Biological Process Terms of DE-mRNAs

**Table S5:** Top20 Cellular Component Terms of DE-mRNAs

**Table S6:** Top20 Molecular Function Terms of DE-mRNAs

**Table S7:** Top20 Cellular Component Terms of the target genes of DE-lncRNAs

**Table S8:** Top20 Biological Process Terms of the target genes of DE-lncRNAs

**Table S9:** The pathway enrich in DE-mRNAm

**Table S10:** The pathway enrich in DE-lncRNAm

## Abbreviations

BAX: BCL2-Associated X
BMP15: bone morphogenetic protein 15
BMPR-1B: bone morphogenetic protein receptor, type 1B
cnci: coding-non-coding index
cpat: coding-potential assessment tool
cpc: coding potential calculator
CTSB: cathepsin B
CTSD: cathepsin D
DE-lncRNA: differentially expressed lncRNA
DE-mRNA: differentially expressed mRNA
E2: estradiol
FDR: false discovery rate
FPKM: fragments per kilobase of exon per million fragments mapped
FSH: follicle-stimulating hormone
GO: gene ontology
HINT2: histidine triad nucleotide binding protein 2
HP: high prolificacy
HRP: horseradish peroxidase
HSPA1A: heat shock protein family A member 1A
HSPA1L: heat shock protein family A member 1 like
Hsp70: heat shock protein family A
KEGG: Kyoto Encyclopedia of Genes and Genomes
KS: *P*-value of Kolmogorov–Smirnov test
LH: luteinizing hormone
lincRNA: long intergennic non-coding RNA
lncRNA: long non-coding RNA
LP: low prolificacy
ncRNA: non-coding RNA
OD: optical density
pfam: protein families database
qRT-PCR: quantitative real-time PCR
ROS: reactive oxygen species
TLR: toll-like receptors
