# Supplementary material for: Genome-wide differential expression profiling of mRNAs and lncRNAs associated with prolificacy in Hu sheep
Source: Biosci Rep. 2018 Apr 27;38(2):BSR20171350. doi: 10.1042/BSR20171350 (PMC5920141; doi:10.1042/BSR20171350)
Supplement: Supplementary file 1 [file bsr20171350_Supp1.pdf]

Supplementary Materials:Genome-wide differential expression profiling of mRNAs and lncRNAs associated with prolificacy in Hu sheep

Xu Feng, Fengzhe Li, Feng Wang, Guomin Zhang, Jing Pang, Caifang Ren,Tingting Zhang, Hua Yang, Ziyu Wang, Yanli Zhang

Table S1. Details of primer sequences, expected product sizes and Genbank accession numbers of genes used for qRT-PCR

Gene	Primer sequence(5′- 3′)	Product size(bp)	Accession number
CCL26	F:GATGTGGCCAAGTTCTGCTG<br>R: TCGGCTGGGCACATACTTTC	148	XM_004020976.3
TSPAN32	F:AAGAAGTGGGTGTGGGCTTT<br>R:CCTGGCCCAATTTGAAGGAC	144	NM_001114667.1
LOC101121216	F:GCCAACTACAAGGGTGCAGA<br>R: TTGTTAGGCAGGCCAGCAG	257	XM_004019478.3
TYROBP	F:ATGCAACTGCTCCTCGTTGA<br>R:GTGATGTGCTGTTTCCGGGT	159	XM_012120538.1
GPNMB	F:CCTGGTATTCCCCAGATGCC<br>R:GTCCACGCTGTCCAGTTGTA	120	XM_004007790.3
TCONS_00025574	F:TGTCCGCTGCTGCCACTTCTA<br>R: TAACGGGCTCCTGCTGGATGAG	132	--
TCONC_00049541	F:TGCTCAGAGGCTCAGTCGTGTT<br>R: CCTCCCAGGCAGTTCAGTGGTA	194	--
TCONC_00530192	F:TGAAGCAGCAGAGGACACAGGT<br>R:AGGTTGCTCAGTGGGTCAGAGG	113	--
TCONS_00560231	F:TGTTGCTGAAGTGGCAGGAACC<br>R:TGTTGCTGAAGTGGCAGGAACC	232	--
TCONC_00920308	F:ACAAGGGAGGCTTGTTGGTG<br>R: CTGAGTCCACTGCTTGGTGA	107	--
BMPR1B	F: GTGCCGTGAACGCACTAACA<br>R: AGACAAAAACGTGCTCCTTCAA	559	NW_011942424.1
CTSB	F: TAGGCTGGGGAGTGGAGAAC<br>R: AGTACTGATGAGTGCACGGC	159	NM_001308587.1
CTSD	F: GCCAGGACCCTGTGTCG<br>R: GCACGTTGTTGACGGAGATG	174	XM_012171958.2
GAPDH	F:GTCAAGGCAGAGAACGGGAA<br>R:GGTTCACGCCCATCACAAAC	232	XM_012166462.1

Table S2 The annotation of all DE-mRNAs

#ID	H1	H2	H3	L5	L7	L8	KEGG_annotation	Swissprot_annotation	nr_annotation
ENSOARG00000000130	4.124731689	3.726211631	4.766965461	1.500059367	1.97862319	2.079188317	K04809 | 0 | oas:101113104 | neuronal acetylcholine receptor subunit alpha-7-like; K04809 nicotinic acetylcholine receptor alpha-7 (A)	Neuronal acetylcholine receptor subunit alpha-7 (Precursor) GN=CHRNA7 OS=Bos taurus (Bovine) PE=2 SV=1	PREDICTED: LOW QUALITY PROTEIN: neuronal acetylcholine receptor subunit alpha-7-like [Ovis aries]
ENSOARG00000001465	3.405431396	0.137444089	1.201888662	5.810695249	7.093629207	5.51918253	--	GTPase IMAP family member 6 GN=GIMAP6 OS=Bos taurus (Bovine) PE=2 SV=1	PREDICTED: GTPase IMAP family member 7-like isoform 1 [Ovis aries]
ENSOARG00000001744	3.690314283	4.312226564	1.386111821	17.41025928	15.29002115	15.02386315	K14378 | 7.4877e-159 | oas:101105447 | TREM2; triggering receptor expressed on myeloid cells 2; K14378 triggering receptor expressed on myeloid cells 2 (A)	Triggering receptor expressed on myeloid cells 1 (Precursor) GN=TREM1 OS=Bos taurus (Bovine) PE=2 SV=1	PREDICTED: triggering receptor expressed on myeloid cells 2 [Ovis aries]
ENSOARG00000001747	9.608946717	9.544950266	5.533109208	16.55068317	16.45007458	18.56610513	K01565 | 0 | oas:101121184 | SGSH; N-sulfoglucosamine sulfohydrolase; K01565 N-sulfoglucosamine sulfohydrolase [EC:3.10.1.1] (A)	Arylsulfatase A (Precursor) GN=ARSA OS=Bos taurus (Bovine) PE=2 SV=1	PREDICTED: N-sulphoglucosamine sulphohydrolase [Ovis aries]
ENSOARG00000002094	45.09405569	48.17747161	21.06712828	88.12350641	87.17829626	115.1314801	K12387 | 1.27089e-176 | oas:101121575 | LAPTM5; lysosomal protein transmembrane 5; K12387 lysosomal-associated transmembrane protein (A)	Lysosomal-associated transmembrane protein 5 GN=LAPTM5 OS=Bos taurus (Bovine) PE=2 SV=1	PREDICTED: lysosomal-associated transmembrane protein 5 [Ovis aries]
ENSOARG00000002862	21.38892612	22.11104804	11.247039	35.56509038	46.84036821	41.66991902	K06545 | 0 | oas:443147 | EM4e; EM4e protein; K06545 CD163 antigen (A)	Soluble CD163 {ECO:0000250 | UniProtKB:Q86VB7} (Precursor) OS=Bos taurus (Bovine) PE=1 SV=2	PREDICTED: LOW QUALITY PROTEIN: uncharacterized protein LOC443147 [Ovis aries]
ENSOARG00000003347	0	0.865299456	0	2.120700616	1.417745551	3.22899783	K17359 | 4.66952e-91 | oas:101120369 | TSPAN32; tetraspanin 32; K17359 tetraspanin-32 (A)	Parotid hormone (Precursor) OS=Sus scrofa (Pig) PE=1 SV=2	PREDICTED: tetraspanin-32 isoform X1 [Bos taurus]
ENSOARG00000003740	10.0528254	9.920952958	9.304096155	5.264629692	3.504580739	3.852621678	K10042 | 0 | oas:101122570 | SUCNR1; succinate receptor 1; K10042 succinate receptor 1 (A)	P2Y purinoceptor 1 GN=P2RY1 OS=Bos taurus (Bovine) PE=2 SV=1	PREDICTED: LOW QUALITY PROTEIN: succinate receptor 1 [Ovis aries]
ENSOARG00000003938	74.48804639	90.10096074	30.34384521	195.4406731	286.9303514	188.9630827	K01379 | 7.61178e-64 | phd:102343491 | CTSD; cathepsin D; K01379 cathepsin D [EC:3.4.23.5] (A)	Cathepsin D (Precursor; Fragment) GN=CTSD OS=Ovis aries (Sheep) PE=1 SV=1	PREDICTED: cathepsin D [Pantholops hodgsonii]
ENSOARG00000004979	73.23556584	91.68820654	25.30948343	177.6899318	263.0103423	175.7671455	K01379 | 3.70961e-65 | lve:103083086 | CTSD; cathepsin D; K01379 cathepsin D [EC:3.4.23.5] (A)	Cathepsin D (Precursor) GN=CTSD OS=Bos taurus (Bovine) PE=1 SV=2	Cathepsin D, partial [Bos mutus]
ENSOARG00000005167	40.58853255	47.26120421	16.01741606	102.4354699	89.21230915	144.5300588	K07992 | 1.50732e-60 | chx:102175151 | TYROBP; TYRO protein tyrosine kinase binding protein; K07992 TYRO protein tyrosine kinase binding protein (A)	TYRO protein tyrosine kinase-binding protein (Precursor) GN=TYROBP OS=Bos taurus (Bovine) PE=3 SV=1	PREDICTED: TYRO protein tyrosine kinase-binding protein isoform X1 [Capra hircus]
ENSOARG00000005236	2.477200372	2.587320056	1.042626099	5.236503019	4.102433766	5.57565066	--	--	PREDICTED: V-set and immunoglobulin domain-containing protein 4-like [Balaenoptera acutorostrata scammoni]
ENSOARG00000006056	0.350541292	0.735692721	0.267072791	0.013869667	0.016362788	0.013772661	K04275 | 0 | phd:102330620 | LPAR4; lysophosphatidic acid receptor 4; K04275 lysophosphatidic acid receptor 4 (A)	Lysophosphatidic acid receptor 5 GN=LPAR5 OS=Bos taurus (Bovine) PE=2 SV=1	PREDICTED: lysophosphatidic acid receptor 4 isoform 1 [Ovis aries]
ENSOARG00000006715	7.029778122	5.851012315	5.239421294	2.885946587	2.434205337	3.053241637	--	Lysophosphatidic acid phosphatase type 6 (Precursor) GN=ACP6 OS=Bos taurus (Bovine) PE=1 SV=1	PREDICTED: acid phosphatase-like protein 2 [Ovis aries]
ENSOARG00000006834	17.24101709	12.50626968	8.129105924	23.02542106	28.31086641	36.89479204	K13625 | 4.45307e-125 | phd:102335976 | ferritin light chain-like; K13625 ferritin light chain (A)	Ferritin light chain GN=FTL OS=Bos taurus (Bovine) PE=2 SV=3	ferritin light chain [Bos taurus]
ENSOARG00000006957	450.7029253	528.9135898	285.9054364	708.5575711	878.7012692	994.7942317	K12382 | 0 | oas:100135695 | PSAP; prosaposin; K12382 saposin (A)	Saposin-D (Precursor) GN=PSAP OS=Bos taurus (Bovine) PE=1 SV=3	PREDICTED: proactivator polypeptide isoform 1 [Ovis aries]
ENSOARG00000006985	38.36290587	41.61754012	16.30831894	61.93929705	66.19518534	79.3853893	K13289 | 0 | oas:101113615 | CTSA; cathepsin A; K13289 cathepsin A (carboxypeptidase C) [EC:3.4.16.5] (A)	Lysosomal protective protein 20 kDa chain (Precursor) GN=CTSA OS=Bos taurus (Bovine) PE=2 SV=1	PREDICTED: lysosomal protective protein [Ovis aries]
ENSOARG00000007110	7.194076423	10.87501055	2.799143814	35.21636177	23.71065745	35.23451252	K06560 | 0 | oas:101111081 | PLA2R1; phospholipase A2 receptor 1, 180kDa; K06560 mannose receptor, C type (A)	Soluble secretory phospholipase A2 receptor (Precursor) GN=PLA2R1 OS=Bos taurus (Bovine) PE=1 SV=1	PREDICTED: secretory phospholipase A2 receptor [Ovis aries]
ENSOARG00000007578	17.12478751	40.81953231	8.509724162	104.7540632	68.67295068	112.3830013	K05768 | 0 | oas:101123369 | SCIN; scinderin; K05768 gelsolin (A)	Adseverin GN=SCIN OS=Bos taurus (Bovine) PE=1 SV=1	PREDICTED: adseverin [Ovis aries]
ENSOARG00000007804	14.83490689	23.44150165	8.486058825	40.56778366	27.82037848	43.29150053	K01300 | 0 | oas:101112329 | CPB2; carboxypeptidase B2 (plasma); K01300 carboxypeptidase B2 [EC:3.4.17.20] (A)	Carboxypeptidase B2 (Precursor) GN=CPB2 OS=Bos taurus (Bovine) PE=1 SV=1	PREDICTED: carboxypeptidase B2 [Ovis aries]
ENSOARG00000007814	4.868073336	5.866350189	1.840311242	10.53162028	9.207296116	10.0162103	K05855 | 0 | oas:101105303 | SYK; spleen tyrosine kinase; K05855 spleen tyrosine kinase [EC:2.7.10.2] (A)	40 kDa tyrosine-protein kinase SYK GN=SYK OS=Sus scrofa (Pig) PE=1 SV=1	Tyrosine-protein kinase SYK, partial [Bos mutus]
ENSOARG00000007932	21.54335293	22.47508683	7.752600196	37.24691933	35.42141401	47.80070975	K18617 | 8.1491e-89 | oas:101121889 | AIF1; allograft inflammatory factor 1; K18617 allograft inflammatory factor 1 (A)	Allograft inflammatory factor 1 GN=AIF1 OS=Bos taurus (Bovine) PE=2 SV=1	PREDICTED: allograft inflammatory factor 1 isoform 1 [Ovis aries]
ENSOARG00000007977	31.62425809	45.50613674	11.7525908	74.229938	76.03323158	92.45617357	K03987 | 8.09548e-111 | oas:101123189 | C1QB; complement component 1, q subcomponent, B chain; K03987 complement C1q subcomponent subunit B (A)	Complement C1q subcomponent subunit B (Precursor) GN=C1QB OS=Bos taurus (Bovine) PE=1 SV=1	PREDICTED: complement C1q subcomponent subunit B [Ovis aries]
ENSOARG00000008001	10.74326797	12.32094882	3.815880836	21.7962624	23.5936009	28.62988712	K03986 | 6.57665e-139 | oas:101102126 | C1QA; complement component 1, q subcomponent, A chain; K03986 complement C1q subcomponent subunit A (A)	Complement C1q subcomponent subunit A (Precursor) GN=C1QA OS=Bos taurus (Bovine) PE=2 SV=1	PREDICTED: complement C1q subcomponent subunit A isoform 1 [Ovis aries]
ENSOARG00000008164	29.82969509	42.59278664	13.22338713	63.58154898	61.63919853	75.44062491	--	Antimicrobial peptide eNAP-1 (Fragment) OS=Equus caballus (Horse) PE=1 SV=1	PREDICTED: LOW QUALITY PROTEIN: granulins [Ovis aries]
ENSOARG00000008167	3.415994637	4.509894769	1.143103314	11.10103531	12.33110595	13.88661218	--	Leukocyte-specific transcript 1 protein GN=LST1 OS=Macaca mulatta (Rhesus macaque) PE=3 SV=1	PREDICTED: leukocyte-specific transcript 1 protein [Ovis aries]
ENSOARG00000008426	0.118828208	0.572024501	0.185908663	3.836512015	1.815296136	4.4616026	K00486 | 0 | oas:101121026 | KMO; kynurenine 3-monooxygenase (kynurenine 3-hydroxylase); K00486 kynurenine 3-monooxygenase [EC:1.14.13.9] (A)	Kynurenine 3-monooxygenase {ECO:0000255 | HAMAP-Rule:MF_03018} OS=Sus scrofa (Pig) PE=2 SV=2	PREDICTED: kynurenine 3-monooxygenase [Ovis aries]
ENSOARG00000008438	48.12028763	56.02308731	17.90598024	89.77885072	75.74290712	95.87426755	K12383 | 1.29262e-113 | oas:101105496 | GM2A; GM2 ganglioside activator; K12383 ganglioside GM2 activator (A)	Ganglioside GM2 activator (Precursor) GN=GM2A OS=Macaca fascicularis (Crab-eating macaque) PE=2 SV=2	PREDICTED: ganglioside GM2 activator [Ovis aries]
ENSOARG00000008861	1.211966985	1.618350264	1.038459151	3.49164358	3.060693063	4.668159976	K16852 | 0 | oas:101120971 | SLAMF6; SLAM family member 6; K16852 SLAM family member 6 (A)	Signaling lymphocytic activation molecule (Precursor) GN=SLAMF1 OS=Canis familiaris (Dog) PE=1 SV=1	PREDICTED: SLAM family member 6 [Ovis aries]
ENSOARG00000008908	9.412649319	11.28054377	5.198960736	19.1647851	24.88969368	23.90837168	K12799 | 2.51557e-141 | oas:101105208 | PYCARD; PYD and CARD domain containing; K12799 apoptosis-associated speck-like protein containing a CARD (A)	Apoptosis-associated speck-like protein containing a CARD GN=PYCARD OS=Bos taurus (Bovine) PE=2 SV=1	PREDICTED: apoptosis-associated speck-like protein containing a CARD isoform 1 [Ovis aries]
ENSOARG00000009000	7.269795274	9.389145972	3.013467114	13.9672554	15.26033789	18.62940135	K06461 | 0 | oas:443217 | ITGAM, C3bi, CD11B, CR3; integrin, alpha M (complement component 3 receptor 3 subunit); K06461 integrin alpha M (A)	Integrin alpha-L (Precursor) GN=ITGAL OS=Bos taurus (Bovine) PE=2 SV=1	integrin alpha-M precursor [Ovis aries]
ENSOARG00000009344	5.187703978	3.201801568	5.530540123	15.56334897	11.62343637	14.24237942	K08753 | 2.75008e-91 | oas:100137067 | FABP4; fatty acid binding protein 4, adipocyte; K08753 fatty acid-binding protein 4, adipocyte (A)	Fatty acid-binding protein, adipocyte GN=FABP4 OS=Cervus elaphus (Red deer) PE=2 SV=1	fatty acid-binding protein, adipocyte [Ovis aries]
ENSOARG00000009626	11.69907459	21.06078071	3.576386533	33.75690634	28.73043988	43.34286664	K06558 | 0 | oas:101121746 | MSR1; macrophage scavenger receptor 1; K06558 macrophage scavenger receptor 1 (A)	Macrophage scavenger receptor types I and II GN=MSR1 OS=Bos taurus (Bovine) PE=1 SV=1	PREDICTED: macrophage scavenger receptor types I and II isoform 1 [Ovis aries]
ENSOARG00000009762	6.127840818	4.683518846	2.455875242	9.677014649	9.103100404	13.40873882	--	Dihydrodiol dehydrogenase 3 OS=Bos taurus (Bovine) PE=2 SV=1	PREDICTED: dihydrodiol dehydrogenase 3-like [Ovis aries]
ENSOARG00000009963	2.192959404	1.922824841	0.519359545	6.820408815	4.91348855	5.463695561	K17310 | 3.19906e-93 | oas:101121216 | serum amyloid A protein-like; K17310 serum amyloid A protein (A)	Amyloid protein A (Precursor) GN=SAA1 OS=Bos taurus (Bovine) PE=1 SV=2	serum amyloid A protein [Bubalus bubalis]
ENSOARG00000011217	5.321972229	6.357966671	1.655969652	11.49720041	9.858819699	14.28442636	K13783 | 0 | oas:101109494 | SLC37A2; solute carrier family 37 (glycerol-3-phosphate transporter), member 2; K13783 MFS transporter, OPA family, solute carrier family 37 (glycerol-3-phosphate transporter), member 1/2 (A)	Sugar phosphate exchanger 2 GN=SLC37A2 OS=Bos taurus (Bovine) PE=2 SV=1	PREDICTED: sugar phosphate exchanger 2 [Ovis aries]
ENSOARG00000011288	3.822256693	5.907407824	5.248680668	12.05024925	10.06275902	9.443023087	K05404 | 0 | chx:102171227 | TLR7; toll-like receptor 7; K05404 toll-like receptor 7 (A)	Toll-like receptor 9 (Precursor) GN=TLR9 OS=Sus scrofa (Pig) PE=2 SV=1	PREDICTED: toll-like receptor 7 isoform X1 [Capra hircus]
ENSOARG00000012307	1.781310087	3.142299727	0.934627269	7.261167162	5.061956052	7.456190044	--	Leucine-rich repeat-containing protein 25 (Precursor) GN=LRRC25 OS=Bos taurus (Bovine) PE=2 SV=1	PREDICTED: LOW QUALITY PROTEIN: leucine-rich repeat-containing protein 25 [Ovis aries]
ENSOARG00000012367	5.081457702	5.609270236	3.153254381	10.76418262	11.06912303	10.60698922	K06464 | 0 | oas:494433 | ITGB2, CD18; integrin, beta 2 (complement component 3 receptor 3 and 4 subunit); K06464 integrin beta 2 (A)	Integrin beta-2 (Precursor) GN=ITGB2 OS=Ovis aries (Sheep) PE=2 SV=1	integrin beta-2 precursor [Ovis aries]
ENSOARG00000012450	132.8153358	107.2708867	52.21277051	191.1758777	202.9396852	224.5781492	K13625 | 4.45307e-125 | phd:102335976 | ferritin light chain-like; K13625 ferritin light chain (A)	Ferritin light chain GN=FTL OS=Bos taurus (Bovine) PE=2 SV=3	ferritin light chain [Bos taurus]
ENSOARG00000012748	27.26841681	46.12732385	21.82752755	80.74261748	95.39571486	88.00608533	--	M-beta (Precursor) GN=PMEL OS=Bos taurus (Bovine) PE=2 SV=2	PREDICTED: transmembrane glycoprotein NMB [Ovis aries]
ENSOARG00000013162	78.66086206	114.2772431	35.12873334	221.1218254	210.3862495	255.6831272	K01369 | 0 | oas:678680 | LGMN; legumain; K01369 legumain [EC:3.4.22.34] (A)	Legumain (Precursor) GN=LGMN OS=Bos taurus (Bovine) PE=1 SV=1	PREDICTED: legumain [Ovis aries]
ENSOARG00000013340	13.35621738	19.40617357	5.798781362	31.01386865	25.40558564	31.02653673	K05514 | 2.17051e-44 | phd:102330755 | CCL26; chemokine (C-C motif) ligand 26; K05514 C-C motif chemokine, other (A)	C-C motif chemokine 26 (Precursor) GN=CCL26 OS=Canis familiaris (Dog) PE=3 SV=1	PREDICTED: c-C motif chemokine 26 [Ovis aries]
ENSOARG00000013445	3.769660409	2.986277426	1.001879507	6.847117429	6.702947055	9.868180117	K10656 | 0 | mcf:102121650 | MARCH1; membrane-associated ring finger (C3HC4) 1, E3 ubiquitin protein ligase; K10656 E3 ubiquitin-protein ligase MARCH1/8 [EC:6.3.2.19] (A)	E3 ubiquitin-protein ligase MARCH8 GN=MARCH8 OS=Bos taurus (Bovine) PE=2 SV=1	E3 ubiquitin-protein ligase MARCH1 [Pteropus alecto]
ENSOARG00000013948	4.242549823	3.094030783	4.937585067	0.610024142	1.784201955	1.905612297	K05175 | 0 | phd:102328547 | GABRA1; gamma-aminobutyric acid (GABA) A receptor, alpha 1; K05175 gamma-aminobutyric acid receptor subunit alpha (A)	Gamma-aminobutyric acid receptor subunit alpha-1 (Precursor) GN=GABRA1 OS=Bos taurus (Bovine) PE=1 SV=1	PREDICTED: gamma-aminobutyric acid receptor subunit alpha-1 isoform 4 [Ovis aries]
ENSOARG00000014339	370.09787	335.0560087	222.3738209	643.3529879	610.0144364	660.1791529	K00522 | 3.73699e-106 | bom:102277133 | FTH1; ferritin, heavy polypeptide 1; K00522 ferritin heavy chain [EC:1.16.3.2] (A)	Ferritin heavy chain, N-terminally processed GN=FTH1 OS=Bos taurus (Bovine) PE=2 SV=3	PREDICTED: ferritin heavy chain [Bos mutus]
ENSOARG00000014440	2.686208646	2.055191655	1.743001177	8.193085693	7.686862951	7.213451515	K08069 | 0 | oas:101105005 | VNN1; vanin 1; K08069 pantetheine hydrolase [EC:3.5.1.92] (A)	Pantetheinase (Precursor) GN=VNN1 OS=Bos taurus (Bovine) PE=2 SV=1	PREDICTED: pantetheinase isoform 1 [Ovis aries]
ENSOARG00000014664	18.85919594	19.53768177	9.299113998	28.1955204	31.35370949	36.8179387	K01366 | 0 | oas:101108427 | CTSH; cathepsin H; K01366 cathepsin H [EC:3.4.22.16] (A)	Cathepsin H light chain (Precursor) GN=CTSH OS=Bos taurus (Bovine) PE=2 SV=1	PREDICTED: pro-cathepsin H [Ovis aries]
ENSOARG00000015082	122.8446698	120.728907	47.45720308	200.0636192	210.4396885	273.7924034	K01052 | 0 | oas:100145859 | LIPA; lipase A, lysosomal acid, cholesterol esterase (EC:3.1.1.13); K01052 lysosomal acid lipase/cholesteryl ester hydrolase [EC:3.1.1.13] (A)	Lysosomal acid lipase/cholesteryl ester hydrolase (Precursor) GN=LIPA OS=Macaca fascicularis (Crab-eating macaque) PE=2 SV=1	lysosomal acid lipase/cholesteryl ester hydrolase precursor [Ovis aries]
ENSOARG00000015263	173.0500573	220.6937075	61.56500466	382.0696052	373.1513633	457.9056218	K01363 | 0 | oas:780470 | CTSB; cathepsin B; K01363 cathepsin B [EC:3.4.22.1] (A)	Cathepsin B heavy chain (Precursor) GN=CTSB OS=Bos taurus (Bovine) PE=1 SV=5	PREDICTED: cathepsin B [Ovis aries]
ENSOARG00000015270	2.049022569	0.902171773	1.402506487	0.221106834	0.208681244	0.373252652	K12026 | 0 | oas:101120134 | TRIM56; tripartite motif containing 56; K12026 tripartite motif-containing protein 56 [EC:6.3.2.19] (A)	E3 ubiquitin-protein ligase TRIM56 GN=TRIM56 OS=Bos taurus (Bovine) PE=3 SV=1	PREDICTED: LOW QUALITY PROTEIN: E3 ubiquitin-protein ligase TRIM56 [Ovis aries]
ENSOARG00000015536	2.036002777	2.265893228	1.009544325	4.058719715	4.723582069	6.236122558	--	Protein O-linked-mannose beta-1,2-N-acetylglucosaminyltransferase 1 GN=POMGNT1 OS=Pongo abelii (Sumatran orangutan) PE=2 SV=1	PREDICTED: protein KIAA1199 homolog [Ovis aries]
ENSOARG00000016127	45.58312483	88.8006795	18.90531177	118.7221103	144.5968568	160.2856782	K08568 | 0 | oas:101101911 | cathepsin Z-like; K08568 cathepsin X [EC:3.4.18.1] (A)	Cathepsin Z (Precursor) GN=CTSZ OS=Bos taurus (Bovine) PE=2 SV=2	PREDICTED: cathepsin Z-like [Ovis aries]
ENSOARG00000017020	21.31976454	26.99365812	7.770026755	67.34632411	48.22976515	66.14823602	K06259 | 0 | oas:101102230 | platelet glycoprotein 4-like; K06259 CD36 antigen (A)	Platelet glycoprotein 4 GN=CD36 OS=Bos taurus (Bovine) PE=1 SV=5	PREDICTED: platelet glycoprotein 4-like [Ovis aries]
ENSOARG00000017314	1.844370475	2.443661484	0.393342424	4.227526125	3.954326334	4.227943653	K00710 | 0 | oas:101114859 | GALNT6; UDP-N-acetyl-alpha-D-galactosamine:polypeptide N-acetylgalactosaminyltransferase 6 (GalNAc-T6); K00710 polypeptide N-acetylgalactosaminyltransferase [EC:2.4.1.41] (A)	Polypeptide N-acetylgalactosaminyltransferase 6 GN=GALNT6 OS=Bos taurus (Bovine) PE=2 SV=1	PREDICTED: polypeptide N-acetylgalactosaminyltransferase 6 [Capra hircus]
ENSOARG00000017679	179.4950699	213.3954176	156.5935899	363.058181	452.0982129	569.3059391	K00522 | 1.10771e-133 | oas:101105484 | ferritin heavy chain-like; K00522 ferritin heavy chain [EC:1.16.3.2] (A)	Ferritin heavy chain, N-terminally processed GN=FTH1 OS=Bos taurus (Bovine) PE=2 SV=3	PREDICTED: ferritin heavy chain-like [Ovis aries]
ENSOARG00000018673	17.7581956	24.57916875	9.817545411	31.33820864	49.66613612	55.29994187	K14379 | 0 | oas:100820744 | ACP5; acid phosphatase 5, tartrate resistant (EC:3.1.3.2); K14379 tartrate-resistant acid phosphatase type 5 [EC:3.1.3.2] (A)	Tartrate-resistant acid phosphatase type 5 (Precursor) GN=ACP5 OS=Sus scrofa (Pig) PE=1 SV=4	tartrate-resistant acid phosphatase type 5 precursor [Ovis aries]
ENSOARG00000019055	3.721860603	4.867734798	1.064155487	8.254057293	6.834899622	8.218539574	K01639 | 0 | oas:101104513 | NPL; N-acetylneuraminate pyruvate lyase (dihydrodipicolinate synthase); K01639 N-acetylneuraminate lyase [EC:4.1.3.3] (A)	N-acetylneuraminate lyase GN=NPL OS=Bos taurus (Bovine) PE=2 SV=1	PREDICTED: N-acetylneuraminate lyase isoform 1 [Ovis aries]
ENSOARG00000019424	6.157482964	10.21456239	4.703646766	23.63948151	23.32290137	25.52034128	K13867 | 0 | oas:101105078 | SLC7A7; solute carrier family 7 (amino acid transporter light chain, y+L system), member 7; K13867 solute carrier family 7 (L-type amino acid transporter), member 7 (A)	Large neutral amino acids transporter small subunit 2 GN=SLC7A8 OS=Pongo abelii (Sumatran orangutan) PE=2 SV=2	PREDICTED: Y+L amino acid transporter 1 isoform 1 [Ovis aries]
ENSOARG00000019463	6.566115342	9.096587093	1.974835136	18.50843398	13.81707174	22.1838928	K06489 | 9.81815e-113 | oas:101112271 | CD53; CD53 molecule; K06489 CD53 antigen (A)	Leukocyte surface antigen CD53 GN=CD53 OS=Bos taurus (Bovine) PE=2 SV=1	CD53 molecule [Ovis aries]
ENSOARG00000019512	1.118294008	1.846332265	0.818428946	3.371887504	2.621860991	2.81561933	--	--	PREDICTED: DENN domain-containing protein 2D [Ovis aries]
ENSOARG00000019535	1.381593682	1.546451635	0.752931477	3.288630052	4.684619187	2.761888297	K12347 | 0 | oas:443365 | SLC11A1, NRAMP1; solute carrier family 11 (proton-coupled divalent metal ion transporters), member 1; K12347 natural resistance-associated macrophage protein (A)	Natural resistance-associated macrophage protein 1 GN=SLC11A1 OS=Ovis aries (Sheep) PE=2 SV=2	natural resistance-associated macrophage protein 1 [Ovis aries]
ENSOARG00000019610	1.971255143	4.161619135	0.815496823	7.775554032	6.710052888	10.54511747	--	--	PREDICTED: transmembrane protein C1orf162 homolog [Ovis aries]
ENSOARG00000019615	6.846834723	8.346089434	3.309882762	22.18984258	14.54363545	21.09159734	--	CMRF35-like molecule 9 (Precursor) GN=CD300LG OS=Bos taurus (Bovine) PE=2 SV=2	PREDICTED: protein ADORA3, isoform 3 isoform X6 [Bubalus bubalis]
ENSOARG00000020511	8.672169123	12.91921723	3.86350624	23.14237141	16.5288691	28.29719942	K10368 | 0 | oas:101101968 | CAPG; capping protein (actin filament), gelsolin-like; K10368 capping protein (actin filament), gelsolin-like (A)	Macrophage-capping protein GN=CAPG OS=Bos taurus (Bovine) PE=2 SV=1	PREDICTED: macrophage-capping protein isoform 1 [Ovis aries]
ENSOARG00000020515	17.1301183	9.322492754	9.02977391	28.08793192	30.03117246	27.89148114	K13915 | 2.26784e-98 | oas:100049062 | LYZ; lysozyme (EC:3.2.1.17); K13915 lysozyme C [EC:3.2.1.17] (A)	Lysozyme C, non-stomach isozyme (Precursor) GN=LYS OS=Bos taurus (Bovine) PE=2 SV=4	lysozyme C precursor [Ovis aries]
ENSOARG00000020667	1.033350017	1.83079845	0.256494546	5.233404163	2.775388697	4.784554793	K06498 | 0 | oas:100192431 | FCGR1A; Fc fragment of IgG, high affinity Ia, receptor (CD64); K06498 high affinity immunoglobulin gamma Fc receptor I (A)	Low affinity immunoglobulin gamma Fc region receptor III (Precursor) GN=FCGR3 OS=Bos taurus (Bovine) PE=2 SV=1	high affinity immunoglobulin gamma Fc receptor I precursor [Ovis aries]
ENSOARG00000020861	153.8533569	198.5161922	73.77632488	308.405971	282.9449019	364.504451	K01368 | 0 | oas:780472 | CTSS; cathepsin S; K01368 cathepsin S [EC:3.4.22.27] (A)	Cathepsin S (Precursor) GN=CTSS OS=Bos taurus (Bovine) PE=1 SV=2	PREDICTED: cathepsin S isoform 1 [Ovis aries]
ENSOARG00000021138	0.047613159	0.06149374	0.051212948	1.265969154	0.800106162	1.077526967	K14538 | 0 | phd:102336213 | GNL3L; guanine nucleotide binding protein-like 3 (nucleolar)-like; K14538 nuclear GTP-binding protein (A)	Guanine nucleotide-binding protein-like 3-like protein GN=GNL3L OS=Bos taurus (Bovine) PE=2 SV=1	PREDICTED: guanine nucleotide-binding protein-like 3-like protein isoform X2 [Capra hircus]
Ovis_aries_newGene_12519	1.015276264	0.950662033	1.24219112	0.012051257	0.298567754	0.011966969	--	LINE-1 reverse transcriptase homolog OS=Nycticebus coucang (Slow loris) PE=3 SV=1	hypothetical protein M91_20604 [Bos mutus]
Ovis_aries_newGene_13826	3.301479169	3.748983373	2.813421785	1.802284195	0.46455905	0.496297476	--	--	PREDICTED: uncharacterized protein LOC101123329 [Ovis aries]
Ovis_aries_newGene_20349	0.024992076	0.075315269	0.008960533	0.166126331	0.223986418	0.416338777	--	--	hypothetical protein M91_10594, partial [Bos mutus]
Ovis_aries_newGene_24826	0.816063288	1.053968791	0.667098886	0.030997162	0.219414055	0.030780363	K10483 | 1.12235e-172 | oas:101103558 | BTBD11; BTB (POZ) domain containing 11; K10483 BTB/POZ domain-containing protein 11 (A)	--	PREDICTED: LOW QUALITY PROTEIN: ankyrin repeat and BTB/POZ domain-containing protein BTBD11 [Ovis aries]
Ovis_aries_newGene_30814	0.236420178	0.244274672	0.050858942	1.885827322	0.847547173	1.605117936	K17914 | 5.61341e-29 | bmor:101735908 | protein split ends-like; K17914 kinesin family member 13 (A)	--	Zinc finger protein 354A, partial [Bos mutus]
Ovis_aries_newGene_36166	0.01926277	0.024878412	0.020719131	0.969466299	0.604235367	0.690227498	--	--	reverse transcriptase-like [Bos taurus]
Ovis_aries_newGene_4861	0.455823621	1.070380152	0.832000707	0.10493273	0.123794752	0.039074556	K08111 | 2.36467e-11 | bom:102284894 | GPC5; glypican 5; K08111 glypican 5 (A)	--	Spectrin beta chain, brain 2 [Bos mutus]

Table S3 Top20 Biological Process Terms of DE-mRNAs

GO.ID	Term	Annotated	Significant	Expected	KS
GO:0009887	organ morphogenesis	2508	5	9.22	3.10E-07
GO:0044763	single-organism cellular process	12940	51	47.59	1.40E-06
GO:0001501	skeletal system development	1683	6	6.19	3.20E-06
GO:0042742	defense response to bacterium	169	7	0.62	3.70E-06
GO:0050896	response to stimulus	9907	50	36.43	1.70E-05
GO:0045654	positive regulation of megakaryocyte differentiation	79	2	0.29	1.90E-05
GO:1901989	positive regulation of cell cycle phase transition	84	1	0.31	2.30E-05
GO:0006958	complement activation, classical pathway	28	2	0.1	2.70E-05
GO:0009888	tissue development	3162	9	11.63	4.50E-05
GO:0044765	single-organism transport	3913	26	14.39	0.00011
GO:0001818	negative regulation of cytokine production	152	6	0.56	0.00026
GO:0002376	immune system process	2537	28	9.33	0.00031
GO:0006950	response to stress	3422	29	12.58	0.00038
GO:0008206	bile acid metabolic process	49	1	0.18	0.00043
GO:0048513	organ development	5586	20	20.54	0.00055
GO:0019882	antigen processing and presentation	106	4	0.39	0.00066
GO:0035095	behavioral response to nicotine	11	1	0.04	0.0008
GO:0071384	cellular response to corticosteroid stimulus	28	1	0.1	0.00084
GO:0050718	positive regulation of interleukin-1 beta secretion	18	1	0.07	0.00087
GO:0016488	farnesol catabolic process	19	1	0.07	0.00098
GO:1900053	negative regulation of retinoic acid	19	1	0.07	0.0009

	biosynthetic process				8
GO:2000224	regulation of testosterone biosynthetic process	19	1	0.07	0.0009 8
GO:0022604	regulation of cell morphogenesis	449	1	1.65	0.0010 6
GO:0042448	progesterone metabolic process	27	1	0.1	0.0012
GO:2000353	positive regulation of endothelial cell apoptotic process	27	1	0.1	0.0012

Annotated: all genes annotated to the function of the number of genes; Significant: different expression gene annotated to the function of the number of genes; Expected: to the function of the number of different expression gene expectations; KS: p value of KS test , the smaller the KS value, indicating its enrichment is more significant.

Table S4 Top20 Cellular Component Terms of DE-mRNAs

GO.ID	Term	Annot	Signifi	Expe	KS

		ated	cant	cted	
GO:0044446	intracellular organelle part	7407	16	27.6	2.70E-05
GO:0005765	lysosomal membrane	98	2	0.37	8.60E-05
GO:0005789	endoplasmic reticulum membrane	601	1	2.24	0.00017
GO:0005576	extracellular region	3294	21	12.27	0.00042
GO:0005892	acetylcholine-gated channel complex	15	1	0.06	0.00043
GO:0005634	nucleus	6087	12	22.68	0.00238
GO:0005938	cell cortex	296	2	1.1	0.00252
GO:0044428	nuclear part	2871	5	10.7	0.00285
GO:0043234	protein complex	4474	15	16.67	0.00347
GO:0030670	phagocytic vesicle membrane	32	1	0.12	0.00644
GO:0097458	neuron part	985	3	3.67	0.00912
GO:0043005	neuron projection	663	2	2.47	0.01114
GO:0005829	cytosol	1316	7	4.9	0.01891
GO:0032991	macromolecular complex	5672	16	21.14	0.0196
GO:0012505	endomembrane system	2949	12	10.99	0.02332
GO:0034364	high-density lipoprotein particle	23	1	0.09	0.02334
GO:0044430	cytoskeletal part	1938	2	7.22	0.02398
GO:0043233	organelle lumen	2404	5	8.96	0.02521
GO:0070013	intracellular organelle lumen	2400	5	8.94	0.02752
GO:0031974	membrane-enclosed lumen	2468	5	9.2	0.03234

Table S5 Top20 Molecular Function Terms of DE-mRNAs

GO.ID	Term	Annotated	Significant	Expected	KS
GO:0005044	scavenger receptor activity	68	2	0.26	2.60E-07
GO:0015464	acetylcholine receptor activity	17	1	0.06	0.00028
GO:0004032	alditol:NADP+ 1-oxidoreductase activity	26	1	0.1	0.00032
GO:0047115	trans-1,2-dihydrobenzene-1,2-diol dehydrogenase activity	20	1	0.08	0.00058
GO:0005506	iron ion binding	212	5	0.81	0.00077
GO:0045703	ketoreductase activity	19	1	0.07	0.00084
GO:0047086	ketosteroid monooxygenase activity	19	1	0.07	0.00084

GO:0047020	15-hydroxyprostaglandin-D dehydrogenase (NADP+) activity	19	1	0.07	0.00084
GO:0047042	androsterone dehydrogenase (B-specific) activity	19	1	0.07	0.00084
GO:0047035	testosterone dehydrogenase (NAD+) activity	19	1	0.07	0.00084
GO:0047006	17-alpha,20-alpha-dihydroxypregn-4-en-3-one dehydrogenase activity	19	1	0.07	0.00084
GO:0045550	geranylgeranyl reductase activity	19	1	0.07	0.00084
GO:0047787	delta4-3-oxosteroid 5beta-reductase activity	19	1	0.07	0.00084
GO:0035410	dihydrotestosterone 17-beta-dehydrogenase activity	19	1	0.07	0.00084
GO:0018636	phenanthrene 9,10-monooxygenase activity	19	1	0.07	0.00084
GO:0047017	prostaglandin-F synthase activity	17	1	0.06	0.00177
GO:0036131	prostaglandin D2 11-ketoreductase activity	17	1	0.06	0.00177
GO:0004252	serine-type endopeptidase activity	158	1	0.6	0.00208
GO:0016787	hydrolase activity	2745	20	10.44	0.0024

Table S6 Top20 Cellular Component Terms of the target genes of DE-lncRNAs

GO.ID	Term	Annotated	Significant	Expected	KS
GO:0050896	response to stimulus	9907	5	4.48	6.50E-07
GO:0044763	single-organism cellular process	12940	6	5.86	1.50E-06
GO:0009888	tissue development	3162	1	1.43	4.40E-05
GO:0044765	single-organism transport	3913	1	1.77	9.70E-05
GO:0006950	response to stress	3422	2	1.55	0.00033
GO:0048513	organ development	5586	2	2.53	0.00039
GO:0044711	single-organism biosynthetic process	1265	1	0.57	0.00124

GO:0051716	cellular response to stimulus	7400	3	3.35	0.00154
GO:0006508	proteolysis	1261	2	0.57	0.00176
GO:0016043	cellular component organization	6252	3	2.83	0.00196
GO:0006810	transport	4832	1	2.19	0.00209
GO:0006957	complement activation, alternative pathway	16	1	0.01	0.00239
GO:0065008	regulation of biological quality	3641	3	1.65	0.00301
GO:0043170	macromolecule metabolic process	9613	6	4.35	0.00375
GO:0045934	negative regulation of nucleobase-containing compound metabolic process	1119	2	0.51	0.00388
GO:0006464	cellular protein modification process	3089	1	1.4	0.00474
GO:0006996	organelle organization	3561	2	1.61	0.0053
GO:0019538	protein metabolic process	5283	4	2.39	0.00546
GO:0002376	immune system process	2537	1	1.15	0.00862
GO:0030154	cell differentiation	4631	2	2.1	0.011
GO:0010466	negative regulation of peptidase activity	174	1	0.08	0.01208
GO:0048869	cellular developmental process	5001	2	2.26	0.01277

Significant: target gene of DE-lncRNAs that annotated to the function of the number of genes

Table S7 Top20 Biological Process Terms of the target genes of DE-lncRNAs

GO.ID	Term	Annotated	Significant	Expected	KS
GO:0044446	intracellular organelle part	7407	6	3.3	2.20E-05
GO:0005765	lysosomal membrane	98	1	0.04	0.00016
GO:0005576	extracellular region	3294	2	1.47	0.00079
GO:0005634	nucleus	6087	5	2.71	0.00218
GO:0044428	nuclear part	2871	4	1.28	0.00304
GO:0043234	protein complex	4474	4	1.99	0.00348
GO:0032991	macromolecular complex	5672	5	2.52	0.0173

					6
GO:0005829	cytosol	1316	1	0.59	0.01898
GO:0012505	endomembrane system	2949	2	1.31	0.02184
GO:0044430	cytoskeletal part	1938	1	0.86	0.02233
GO:0043233	organelle lumen	2404	3	1.07	0.02791
GO:0070013	intracellular organelle lumen	2400	3	1.07	0.03044
GO:0031300	intrinsic component of organelle membran...	167	0	0.07	0.03089
GO:0044422	organelle part	7655	6	3.41	0.03246
GO:0031974	membrane-enclosed lumen	2468	3	1.1	0.03541
GO:0036128	CatSper complex	14	1	0.01	0.03742
GO:0031981	nuclear lumen	2071	2	0.92	0.03743
GO:0015630	microtubule cytoskeleton	1336	1	0.59	0.04631
GO:0005694	chromosome	909	1	0.4	0.04749
GO:0034707	chloride channel complex	41	1	0.02	0.05312

Table S8 The pathway enrich in DE-mRNAs

**#Kegg_pathway**	**ko_id**	**Cluter_frequency**	**P-value**	**Corrected_P-value**
Lysosome	ko04142	14 out of 47 29.7872340425532%	4.24E-14	1.40E-12
Tuberculosis	ko05152	8 out of 47 17.0212765957447%	2.59E-05	0.000854
Mineral absorption	ko04978	5 out of 47 10.6382978723404%	3.46E-05	0.001141
Staphylococcus aureus infection	ko05150	5 out of 47 10.6382978723404%	0.00011	0.003615
Pertussis	ko05133	5 out of 47 10.6382978723404%	0.000116	0.003835
Phagosome	ko04145	7 out of 47 14.8936170212766%	0.000178	0.005882
Osteoclast differentiation	ko04380	5 out of 47	0.0007	0.025813

		10.6382978723404%	82	
Complement and coagulation cascades	ko04610	3 out of 47 6.38297872340426%	0.011377	0.375438
Leishmaniasis	ko05140	3 out of 47 6.38297872340426%	0.013024	0.429805
Legionellosis	ko05134	3 out of 47 6.38297872340426%	0.014345	0.473391
Hematopoietic cell lineage	ko04640	3 out of 47 6.38297872340426%	0.017714	0.584572
Prion diseases	ko05020	2 out of 47 4.25531914893617%	0.018636	0.614976
Fc gamma R-mediated phagocytosis	ko04666	3 out of 47 6.38297872340426%	0.019282	0.636317
Antigen processing and presentation	ko04612	3 out of 47 6.38297872340426%	0.024436	0.8064
Nicotine addiction	ko05033	2 out of 47 4.25531914893617%	0.028633	0.944905
Natural killer cell mediated cytotoxicity	ko04650	3 out of 47 6.38297872340426%	0.030956	1
Systemic lupus erythematosus	ko05322	3 out of 47 6.38297872340426%	0.051747	1
Malaria	ko05144	2 out of 47 4.25531914893617%	0.055021	1
PPAR signaling pathway	ko03320	2 out of 47 4.25531914893617%	0.066272	1
Rheumatoid arthritis	ko05323	2 out of 47 4.25531914893617%	0.143866	1
Rap1 signaling pathway	ko04015	3 out of 47 6.38297872340426%	0.146046	1
Regulation of actin cytoskeleton	ko04810	3 out of 47 6.38297872340426%	0.151728	1
Protein digestion and absorption	ko04974	2 out of 47 4.25531914893617%	0.162786	1
Sphingolipid signaling pathway	ko04071	2 out of 47 4.25531914893617%	0.167053	1
Leukocyte transendothelial migration	ko04670	2 out of 47 4.25531914893617%	0.169193	1
Chagas disease (American trypanosomiasis)	ko05142	2 out of 47 4.25531914893617%	0.169193	1
Amoebiasis	ko05146	2 out of 47 4.25531914893617%	0.17349	1
Neuroactive ligand-receptor interaction	ko04080	3 out of 47 6.38297872340426%	0.250528	1
Influenza A	ko05164	2 out of 47	0.2979	1

		4.25531914893617%	94	
Transcriptional misregulation in cancer	ko05202	2 out of 47<br>4.25531914893617%	0.3114<br>32	1
Cell adhesion molecules (CAMs)	ko04514	2 out of 47<br>4.25531914893617%	0.3270<br>52	1
Viral carcinogenesis	ko05203	2 out of 47<br>4.25531914893617%	0.4183<br>88	1
PI3K-Akt signaling pathway	ko04151	2 out of 47<br>4.25531914893617%	0.6561<br>37	1

Table S9 The pathway enrich in DE-lncRNAs

**#Kegg_pathway**	**ko_id**	**Cluter_frequency**	**P-valu e**	**Corrected_P-va lue**
Spliceosome	ko03040	2 out of 6<br>33.3333333333333%	0.0072<br>33	0.13743
Other glycan degradation	ko00511	1 out of 6<br>16.6666666666667%	0.0205<br>97	0.391341
Aminoacyl-tRNA biosynthesis	ko00970	1 out of 6<br>16.6666666666667%	0.0363<br>71	0.691052
Sphingolipid metabolism	ko00600	1 out of 6<br>16.6666666666667%	0.0371<br>17	0.705223
Lysine degradation	ko00310	1 out of 6<br>16.6666666666667%	0.0511<br>97	0.972741
Complement and coagulation cascades	ko04610	1 out of 6<br>16.6666666666667%	0.0585<br>38	1
Staphylococcus aureus infection	ko05150	1 out of 6<br>16.6666666666667%	0.0600<br>01	1

RNA degradation	ko03018	1 out of 6 16.6666666666667%	0.0600 01	1
Legionellosis	ko05134	1 out of 6 16.6666666666667%	0.0636 49	1
Estrogen signaling pathway	ko04915	1 out of 6 16.6666666666667%	0.0759 64	1
Antigen processing and presentation	ko04612	1 out of 6 16.6666666666667%	0.0774 04	1
Lysosome	ko04142	1 out of 6 16.6666666666667%	0.0952 47	1
Toxoplasmosis	ko05145	1 out of 6 16.6666666666667%	0.1008 95	1
Measles	ko05162	1 out of 6 16.6666666666667%	0.1030 06	1
Influenza A	ko05164	1 out of 6 16.6666666666667%	0.1314 39	1
Protein processing in endoplasmic reticulum	ko04141	1 out of 6 16.6666666666667%	0.1362 17	1
Epstein-Barr virus infection	ko05169	1 out of 6 16.6666666666667%	0.1564 5	1
Endocytosis	ko04144	1 out of 6 16.6666666666667%	0.1683 98	1
MAPK signaling pathway	ko04010	1 out of 6 16.6666666666667%	0.1867 04	1
